# Postoperative Intravenous Iron Infusion in Anemic Colorectal Cancer Patients: An Observational Study

**DOI:** 10.3390/biomedicines12092094

**Published:** 2024-09-13

**Authors:** Leonidas Chardalias, Androniki-Maria Skreka, Nikolaos Memos, Alexandra-Stavroula Nieri, Dimitrios Politis, Marianna Politou, Theodosios Theodosopoulos, Ioannis Papaconstantinou

**Affiliations:** 1 2nd Surgical Department, Aretaieion University Hospital, National and Kapodistrian University of Athens, 11528 Athens, Greece; 2 Department of Nursing, National and Kapodistrian University of Athens, 11527 Athens, Greece; alexandranieri@hotmail.gr; 3 Hematology Laboratory-Blood Bank, Aretaieion Hospital, National and Kapodistrian University of Athens, 11528 Athens, Greece

**Keywords:** colorectal cancer, anemia, intravenous iron, ferric carboxymaltose, postoperative anemia

## Abstract

Anemia is the most common extraintestinal symptom of colorectal cancer, with a prevalence of 30–75%. While the preoperative anemia in this patient population has been well studied and its correction 4–6 weeks prior to surgery is recommended when feasible, there is a paucity of data regarding the management of postoperative anemia, which has a prevalence of up to 87% in these patients. To address this issue, we conducted an observational cohort study of surgically treated postoperative anemic patients with colorectal cancer. The objective of this study was to evaluate the effect of intravenous ferric carboxymaltose on the correction of postoperative anemia by postoperative day 30 (POD30). The primary outcome was the change in hemoglobin on POD30, while the secondary outcomes were the change in iron and other laboratory parameters, postoperative complications and transfusions. The results demonstrated that patients treated with intravenous iron exhibited a significant increase in hemoglobin levels by POD30, along with a concomitant increase in hematocrit, ferritin, and transferrin saturation levels, compared to the control group. The findings imply that patients undergoing colorectal cancer surgery with anemia that was not corrected in the preoperative setting may benefit from early postoperative intravenous iron infusion.

## 1. Introduction

Colorectal cancer (CRC) is the third-most commonly occurring cancer worldwide and the second in cancer-related deaths [1]. Anemia is present in 30–75% of CRC patients, with iron deficiency being the main culprit in 88% of cases followed by functional iron deficiency due to inflammation [2,3,4]. The presence of iron deficiency anemia (IDA) in CRC patients has been linked to an increased requirement for transfusions, elevated rates of morbidity, and even a worse prognosis [5,6].

Erythropoietin has been used to treat anemia in CRC, but it has been associated with higher rates of tumor recurrence and a poorer prognosis. Consequently, it is not currently recommended as a course of action [7,8]. The standard-of-care management for anemia in this patient population has traditionally involved administration of oral or intravenous iron.

Daily per os iron supplementation, still widely used today despite its economic advantages, is associated with gastrointestinal side effects, poor patient compliance, poor absorption and an inability to effectively be used in inflammatory situations due to the hepcidin pathway [9]. On the other hand, intravenous iron has emerged as the gold standard as it necessitates a single or double 15 min infusion, yields superior outcomes in terms of hemoglobin (Hb) elevation, and bypasses the gastrointestinal tract, thereby averting the hepcidin-mediated inhibition of iron absorption in the duodenum [10].

Some studies emphasize the need for preoperative correction of anemia to avoid perioperative transfusions and reduce the incidence of postoperative complications [11]. It is evident that the administration of iron therapy four to six weeks prior to surgery, as observed in the majority of clinical trials, presents a significant challenge in terms of logistical coordination and patient adherence. A randomized controlled trial (RCT) on preoperative iron infusion versus preoperative per os iron supplementation 2 weeks before a surgical operation showed that the hemoglobin was not normalized prior to surgery, but at subsequent time points [12].

There is a paucity of studies examining the postoperative management of anemia in CRC patients. Postoperative CRC anemia is a frequent finding at the time of discharge, with up to 87% of patients leaving the hospital anemic [13,14]. A recent RCT involving a mixed population (orthopedic, gynecologic, general surgery) demonstrated the superiority of intravenous (IV) iron over standard care in the management of postoperative anemia, but it lacked focus on CRC patients [15]. The only retrospective study specifically evaluating the impact of postoperative iron administration in CRC patients found that iron improved the recovery of hemoglobin by POD30 without increasing postoperative complications [16].

To further investigate this gap in the literature on postoperative IV iron treatment in CRC surgical patients with anemia, we conducted an observational cohort study of this group of patients in our department. A comparison was made between two groups of anemic CRC surgical patients on POD3: those who received IV iron on the same day and those who received routine postoperative care without oral or IV iron supplementation. The primary outcome was the change in hemoglobin on postoperative day 30. Secondary endpoints included iron parameters, postoperative complications, transfusions, and other laboratory values. The aim of this study was to add evidence to the limited data on the management of postoperative anemia in CRC patients.

## 2. Materials and Methods

We conducted a prospective, observational cohort study at the 2nd Surgical Department of Aretaieion University Hospital of Athens, a tertiary center for colorectal cancer. The hospital ethics committee approved the trial (approval code 401/09-02-2022, approval date 9 February 2022).

Eligible participants were individuals aged over 18 years who had undergone elective surgery for non-metastatic colorectal cancer and were anemic on POD3, as determined by routine laboratory exams. A blood sample was taken preoperatively (one or a few days before surgery) on POD3 and on POD30 according to routine practice in our hospital.

We included participants with a hemoglobin concentration between 70 g/dL and 120 g/dL for women and 70 g/dL and 130 g/dL for men, in accordance with the World Health Organization’s definition of anemia. With this approach, we aimed to include patients with clinically significant anemia and to ensure consistency with previous studies of iron supplementation in surgical patients [17]. Participants treated with intravenous ferric carboxymaltose at a dose of 15 mg/kg (Ferinject, Vifor Pharma, Paris, France) at the discretion of the responsible physician were classified as the intravenous iron (IVI) group. The maximum dose was 1000 mg administered over 15–20 min. Although the total cumulative dose may reach 2000 mg, the maximum dose that can be safely administered over a seven-day period is 1000 mg. Therefore, our dosing regimen was designed to target the maximum dose during the patient’s postoperative stay, which is typically less than one week, without necessitating a second outpatient visit. The control group received routine postoperative care without per os or IV iron supplementation. Patients were assigned to either treatment based on the clinical judgement of the treating physicians. The decision to administer IV iron was guided primarily by the hemoglobin levels (the range mentioned above), but also by the iron parameters when available (transferrin saturation (TSAT) < 20% or ferritin < 100 ng/mL) or the clinical status of the patients. Both groups were monitored, and blood transfusions were administered when clinically indicated as per the treating physician’s discretion.

Based on these values, a comparison was made between the two groups in respect of the change in hemoglobin levels and other laboratory values related to anemia. The primary marker employed was hemoglobin, as it is the most direct measure of anemia and a key determinant that drives the surgeon’s decisions regarding the patient’s management. Furthermore, hematocrit (HCT) and red blood cell (RBC) counts were included in the assessment of the effects of IV iron on blood volume and erythrocyte production. The iron biomarker ferritin was employed as an indicator of total body iron stores whereas transferrin saturation (TSAT) reflects the proportion of transferrin that is saturated with iron, indicating immediate iron availability. Both of these were utilized to define iron deficiency and to evaluate the response to iron therapy. In addition, data such as the number of transfusions, 30-day readmission rate, and adverse effects of iron transfusion were compared between the two groups.

Patients were excluded from the analysis if they had Clavien–Dindo complications of grade IIIb or above, had a chronic kidney disease of grade III or above, had a history of acquired iron overload, or had a family history of hemochromatosis or thalassemia, a known cause of anemia (e.g., untreated B12 or folate deficiency or hemoglobinopathy). Patients with a temperature exceeding 37.5 °C or those taking non-prophylactic antibiotics on POD3, as well as patients who had received erythropoietin or iron therapy, either orally or intravenously, in the previous 12 weeks, were on immunosuppressive therapy (for organ transplantation), were pregnant, or were breastfeeding were excluded from the analysis.

The decision regarding the necessity for intraoperative blood transfusion is made in accordance with the established guidelines for acute blood loss and depends on the counted volume of blood loss [18]. In the postoperative period, the American Society of Anesthesiology guidelines were followed [19]. Transfusion in hemodynamically stable patients is triggered by a limit of Hb < 7.0 g/dL, with the exception of acute coronary events, where the desired levels of hemoglobin are 9–10 g/dL [20]. In all cases, the decision to transfuse was based on the patient’s clinical status [21,22].

### Statistical Analysis

Data analysis was performed using the statistical software IBM SPSS Statistics for Windows, Version 28.0. Armonk, NY, USA: IBM Corp. The level of statistical significance was set at a = 0.05. Firstly, a descriptive statistical analysis was conducted. Qualitative variables are presented as either total (Ν) or relative (%) frequencies. Quantitative variables are presented as either mean and standard deviation (SD) or as median and interquartile range (IQR) (25th–75th percentile), depending on whether a normal distribution was observed. The histogram of each variable in conjunction with the Shapiro–Wilk test was employed to ascertain its normal distribution. To compare the demographic characteristics, clinical data, and laboratory values between the two groups, the independent sample *t*-test was employed for continuous variables, the Mann–Whitney was used for variables that did not follow a normal distribution and the chi-squared test (or Fisher’s exact test or Monte Carlo test if the criteria for the chi-squared test were not met) was used for qualitative variables. Additionally, a two-way repeated-measure analysis of variance (ANOVA) was used to compare the hematological markers between the two groups at the three designated time points, provided that the data exhibited a normal distribution. In cases where the hematological markers did not follow a normal distribution, the Friedman test, a non-parametric alternative to the one-way ANOVA with repeated measures, was employed. Following the rejection of the null hypothesis of an initial two-way repeated-measure ANOVA or Friedman test, a post hoc test was conducted using the Bonferroni test.

The median percentage change in a laboratory value was defined as: ((POD30 value-POD3 value)/POD3 value) × 100. Comparisons were conducted separately for men and women, given the existence of disparate hemoglobin levels in the two sexes corresponding to anemia.

## 3. Results

The initial study cohort comprised 104 patients. However, eight patients were unable to be contacted for follow-up, four patients experienced severe complications or death, seven patients were found to have metastatic disease, five patients had benign lesions, and two patients received postoperative transfusions and were excluded from the study.

The final study sample comprised 78 patients who exhibited anemia on POD3 following surgery for CRC. Of the aforementioned patients, 36 received intravenous iron (Ferinject) on POD3, while the remaining 42 did not receive IV iron and constituted the control group.

### 3.1. Baseline Characteristics and Main Outcomes

The two groups were found to have comparable baseline characteristics. The IV group exhibited a significantly elevated incidence of postoperative complications in comparison to the control group (type I–IIIa): 38.9% vs. 19.0% (*p* = 0.041). The majority of these complications were classified as type I, as detailed in Table 1. In addition, patients in the IV iron group had a longer length of hospital stay (1 day) than those in the control group (*p* = 0.011).

The two groups had no differences in intraoperative blood loss, the number of intraoperative transfusions, the readmission rate, or other key outcomes. These findings are presented in Table 1.

No serious adverse effects related to ferric carboxymaltose infusion were recorded.

### 3.2. Hemoglobin Levels

The median (IQR) percentage increase in hemoglobin from POD3 to POD30 was 3.58% (2.22–4.79%) in the control group and 5.93% (3.63–9.35%) in the IV iron group. The percentage increase in hemoglobin from POD3 to POD30 was statistically significantly greater in the IV iron group than in the control group (*p* = 0.002). In particular, in men, the median (IQR) percentage increase in hemoglobin from POD3 to POD30 was 2.44% (1.35–4.22%) in the control group and 5.59% (3.69–9.79%) in the IV iron group (*p* = 0.004). In women, the median (IQR) percentage increase in hemoglobin from POD3 to POD30 was 3.79% (1.67–5.56%) in the control group and 6.11% (2.55–7.58%) in the IV iron group (*p* = 0.107) (Table 2).

In males, no significant differences were observed in preoperative hemoglobin levels between the IV iron group and the control group (*p* = 0.387) or on POD3 (*p* = 0.303) or POD30 (*p* = 0.309). The hemoglobin levels at POD3 were significantly lower in comparison to the preoperative levels in both groups (control group: mean difference (95% CI): −1.85 (−2.53, −1.18) g/dL, *p* < 0.001; IV iron group: (mean difference (95% CI): −1.84 (−2.48, 1.20) g/dL, *p* < 0.001).

**Figure 1:**
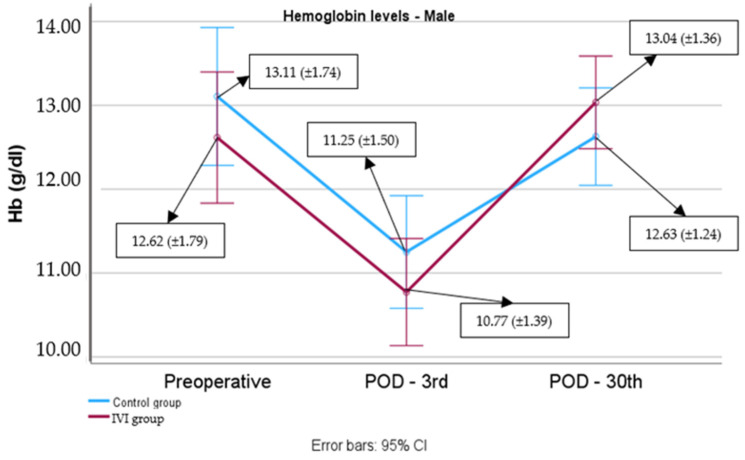
Hemoglobin levels from preoperative to POD30 in males.

The hemoglobin levels in men at POD30 were significantly higher than those at POD3 levels in the control group (mean difference (95% CI): 1.38 (0.72, 2.03) g/dL, *p* < 0.001), as well as in the IV iron group (mean difference (95% CI): 2.26 (1.16, 2.88) g/dL, *p* < 0.001). The increase in hemoglobin levels from POD3 to POD30 was statistically significantly greater in the IV iron group compared to the control group in the male cohort (mean difference (95% CI): 0.89 (0.17, 1.61), *p* = 0.018) (Figure 1).

In females, there were no significant differences in preoperative (*p* = 0.680) and POD3 (*p* = 0.256) hemoglobin levels between the IV iron group and the control group. However, on POD30, women in the IV iron group exhibited significantly elevated hemoglobin levels than those of the control group (*p* = 0.042).

**Figure 2:**
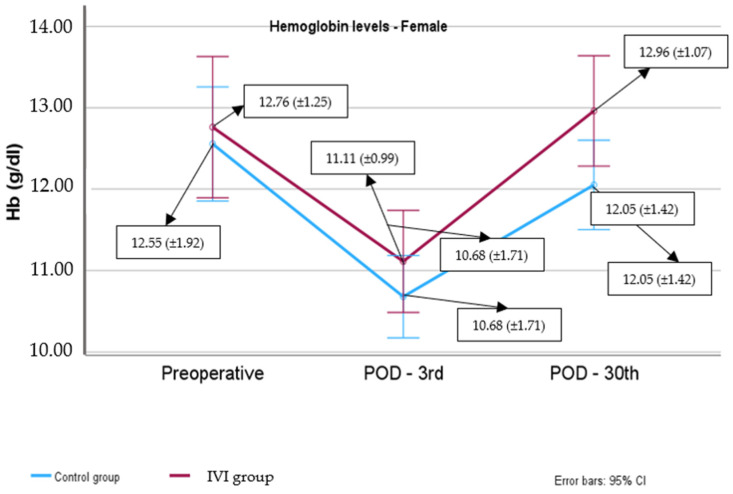
Hemoglobin levels from preoperative to postoperative day 30, female.

The hemoglobin levels in women at POD3 were significantly lower compared to the preoperative levels in the control group (mean difference (95% CI): −1.88 (−2.45, −1.30) g/dL, *p* < 0.001) as well as in the IV iron group (mean difference (95% CI): −1.65 (−2.37, −0.93) g/dL, *p* < 0.001). Hemoglobin levels at POD30 were significantly higher compared to POD3 levels in the control group (mean difference (95% CI): 1.37 (0.82, 1.93) g/dL, *p* < 0.001), as well as in the IV iron group (mean difference (95% CI): 1.85 (1.16, 2.53) g/dL, *p* < 0.001). Finally, the increase in hemoglobin levels from POD3 to POD30 was comparable between the two groups in the female cohort (*p* = 0.184) (Figure 2).

### 3.3. Hematocrit Levels

With regard to HCT, the median (IQR) percentage increase in HCT levels from POD3 to POD30 was significantly greater in the IV iron group compared to the control group (18.70% (9.05–27.69%) vs. 10.77% (6.59–19.07%), *p* = 0.031). In particular, the median (IQR) percentage increase in HCT levels from POD3 to POD30 in males was 11.80% (6.38–18.52%) in the control group and 20.27% (11.75–36.58%) in the IV iron group (*p* = 0.029). Correspondingly, in women, the median (IQR) percentage increase in HCT levels from POD3 to POD30 was 10.55% (6.67–21.48%) in the control group and 15.76% (5.22–24.00%) in the IV iron group (*p* = 0.516).

### 3.4. RBC Counts

**Figure 3:**
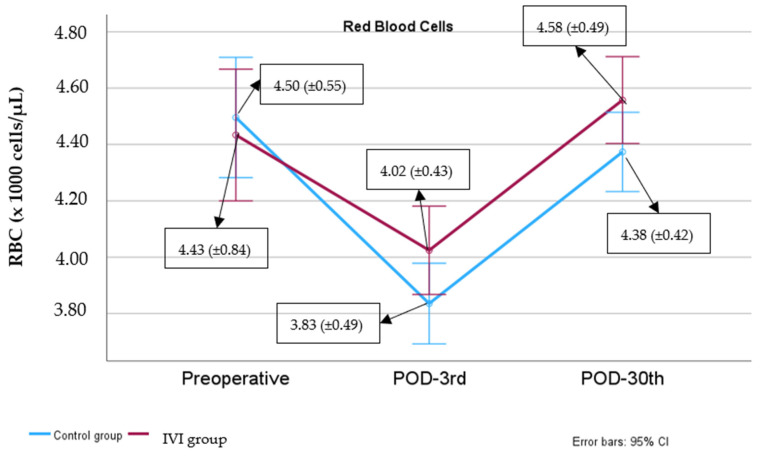
Red blood cell levels from preoperative to postoperative day 30.

The RBC count was comparable between the two groups preoperatively (*p* = 0.781) and on POD3 (*p* = 0.163) and POD30 (*p* = 0.083). In both groups, the erythrocyte count on POD3 was significantly lower than preoperatively (mean difference (95% CI): −0.66 (−0.88, −0.44) × 1000 cells/μL, *p* < 0.001 in the control group and −0.41 (−0.65, −0.16) × 1000 cells/μL, *p* < 0.001 in the IVI group). On POD30, the RBC count was significantly higher than on POD3 (mean difference (95% CI): 0.54 (0.50, 0.58) × 1000 cells/μL, *p* < 0.001 in the control group and 0.53 (0.49, 0.57) × 1000 cells/μL, *p* < 0.001 in the IVI group). The median percentage change (IQR) in RBC counts in the control group was 13.99% (12.23–17.23%) while in the IVI group it was 13.11% (12.26–13.93%) (*p* = 0.145). (Figure 3).

### 3.5. Ferritin Levels

**Figure 4:**
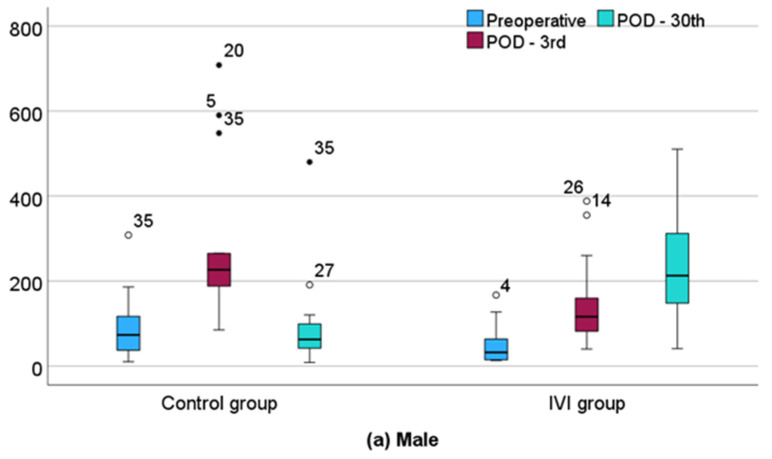

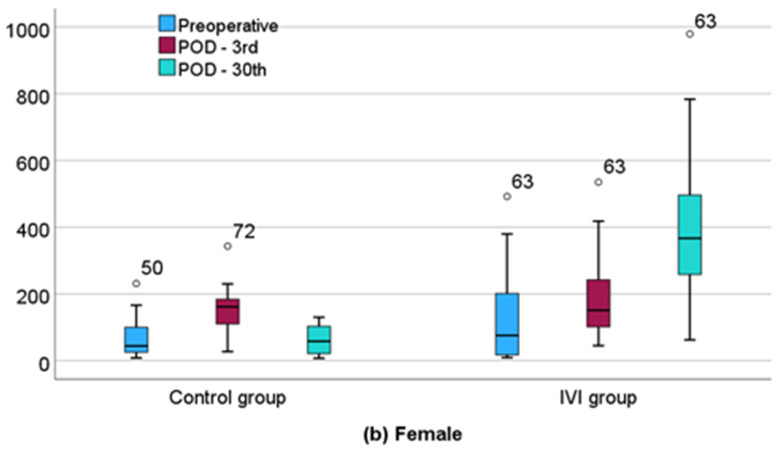
(**a**) Ferritin levels from preoperative to postoperative day 30 in the male cohort. (**b**) Ferritin levels from preoperative to postoperative day 30 in the female cohort. The full black circle is an indication that an extreme outlier is present in the data. The circle is an indication that an outlier is present in the data.

The median (IQR) percentage change in ferritin levels from POD3 to POD30 was −69.97% (−85.14%, −52.86%) in the control group and 109.61% (18.66%, 215.54%) in the IVI group (*p* < 0.001). Specifically, in males, the median (IQR) percentage change in ferritin from POD3 to POD30 was −74.32% (−84.77%, 62.26%) in the control group and 79.41% (1.08%, 203.29%) in the IVI group (*p* < 0.001). Furthermore, in women, the median (IQR) percentage increase in ferritin from POD3 to POD30 was −68.47% (−85.36%, −37.68%) in the control group and 176.56% (58.17%, 261.33%) in the IVI group (*p* < 0.001). The ferritin levels in both groups are presented separately for males and females in Figure 4a,b.

### 3.6. CRP Levels

There was no statistically significant difference in CRP levels between the two groups preoperatively (*p* = 0.771), on POD3 (*p* = 0.988), or POD30 (*p* = 0.667). A significant increase was observed in both groups on POD3 compared to the preoperative value (control group: 8.39 (5.94, 10.83), *p* < 0.001; IV iron group: 7.27 (4.62, 9.92), *p* < 0.001). On POD30, CRP was similar to that preoperatively.

### 3.7. TSAT Levels

There was no significant difference in TSAT levels between the two groups preoperatively (*p* = 0.873) or at POD3 (*p* = 0.476). However, patients in the IVI group exhibited significantly elevated TSAT levels at POD30 compared to the control group (*p* < 0.001). In the control group, TSAT levels on POD3 (median (IQR): 14.19 (10.47, 20.00)) were significantly lower than preoperative TSAT levels (27.21 (18.92, 36.44)) (*p* = 0.001). Correspondingly, POD30 TSAT levels (20.05 (11.71, 29.14)) were significantly lower than the preoperative ones (*p* = 0.045). TSAT levels did not differ significantly between POD3 and POD30 levels (*p* = 0.637).

**Figure 5:**
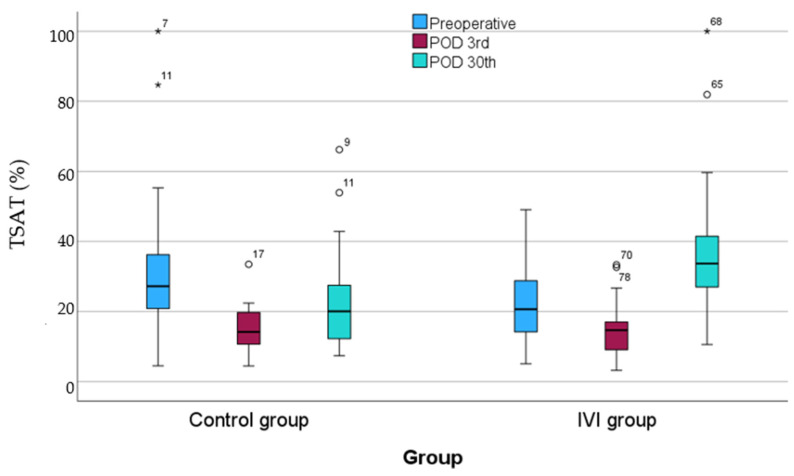
TSAT levels from preoperative to postoperative day 30. The asterisk (*) is an indication that an extreme outlier is present in the data. The circle is an indication that an outlier is present in the data.

Regarding the IVI group, TSAT levels on POD3 (14.66 (8.2, 17.33)) were significantly lower than preoperative TSAT levels (20.65 (12.58, 29.60)) (*p* = 0.009). Conversely, POD30 TSAT levels (33.67 (26.91, 41.95)) were significantly higher than preoperative levels (*p* = 0.002), as well as those of POD3 (*p* < 0.001) (Figure 5).

### 3.8. Additional Laboratory Examinations

As detailed in Appendix A, platelet, folic acid, vitamin B12, international normalized ratio (INR), fibrinogen, and activated partial thromboplastin time (APTT) levels were recorded at each observation time point. There were no statistically significant differences between the two groups with respect to these values.

### 3.9. Iron Deficiency

Complete data for the iron parameters (Fe, TIBC and ferritin) were available for 32 of the 42 patients in the control group and 34 of the 36 patients in the IVI group. A subgroup analysis was conducted (Table 3).

In the control group, 24 patients (75%) exhibited iron deficiency, whereas in the IVI group, 29 patients (85.3%) had iron deficiency. The proportion of patients with iron deficiency did not differ significantly between the two groups (*p* = 0.293).

In patients without iron deficiency, the percentage change in hemoglobin did not differ significantly between the two groups (*p* = 0.622). However, in patients with iron deficiency, the percentage change in hemoglobin was significantly greater in the IVI group compared to the control group (*p* = 0.006).

The percentage change in ferritin was found to be statistically significantly different between the IVI group and the control group in both iron-deficient (*p* < 0.0001) and non-iron-deficient patients (*p* = 0.002).

TSAT levels on POD3 did not differ significantly between the IVI group and the control group, neither in the iron-deficient patients (*p* = 0.284) nor in the patients without iron deficiency (*p* = 0.861). On POD30, TSAT levels were significantly higher in the IVI group than in the control group, both in patients without iron deficiency (*p* = 0.030) and in those with iron deficiency (*p* < 0.001).

## 4. Discussion

Preoperative anemia in colorectal cancer patients is a common finding and is linked to a greater number of perioperative transfusions, poorer postoperative outcomes, and a worse prognosis [23]. Many studies have been conducted on the necessity for correction of anemia in the preoperative setting. A recent systematic review and meta-analysis of 465 patients (three RCTs) [11] demonstrated that preoperative correction of anemia (4–6 weeks prior to surgery) with intravenous iron resulted in a statistically significant reduction in the risk of blood transfusion and a significant increase in hemoglobin levels on index admission. These results further support the patient blood management recommendations, which advocate for the preoperative administration of iron supplementation to patients undergoing elective surgery with the aim of reducing the necessity for perioperative transfusions [24]. However, particularly due to the COVID-19 pandemic, patients are presenting with more symptomatic or advanced cancers and the time to surgery is frequently less than the well-studied 4- to 6-week window [25,26].

The necessity of correcting postoperative anemia in CRC surgery has not been sufficiently addressed in the existing literature. International guidelines highlight the importance of correcting anemia, either preoperatively or until complete recovery from surgery [27]. Postoperative CRC anemia is a common occurrence at the time of discharge, with up to 87% of patients leaving the hospital anemic [13,14]. It is significantly associated with increased rates of RBC transfusion, prolonged hospital stay, and poorer quality of life and overall survival [14]. The combination of preoperative and postoperative anemia in this group of patients further worsens their outcomes [28].

The objective of this prospective observational study was to evaluate the efficacy of intravenous iron infusion in correcting postoperative anemia. To our knowledge, this is the first prospective study to assess the efficacy of IV iron in the treatment of postoperative anemia in CRC patients with a comparison of IV iron therapy to no iron treatment. An important randomized controlled trial compared postoperative IV iron infusion with that of standard care [15]. The results demonstrated that surgical patients with functional iron deficiency on POD1 who were treated with a single dose of 1 g of IV Ferinject, had significantly higher hemoglobin levels (mean difference of 7.84 g/L, 95% CI 3.79–11.9; *p* < 0.0001) along with elevated iron, iron saturation, and ferritin levels, compared to the standard care (observation) group. These patients also received significantly fewer blood units than the controls. However, the cohort of patients under investigation was heterogeneous, with the majority comprising orthopedic surgical patients. Only 19 patients who had undergone abdominal surgery were included in the study. The only study that has focused on this specific group of patients (CRC) is a retrospective study comparing postoperative IV iron administration versus standard care [16]. The study compared CRC surgical patients who were anemic on POD1 and either received IV iron sucrose treatment postoperatively (in multiple doses) or were in the standard care group (oral or no iron at all). The IV iron group had statistically significantly lower hemoglobin levels on the day of surgery and at POD1. The results showed that 72% of patients in both groups remained anemic at POD30. Furthermore, the IV iron group had a more pronounced increase in hemoglobin levels by POD30 compared to the standard care group.

The present study identifies several important findings pertaining to the administration of IV iron in colorectal cancer patients. Firstly, it was demonstrated that the hemoglobin levels at POD3 were predictably lower than the preoperative levels, primarily due to surgical factors including blood loss, inflammation, and fluid shifts. Despite that, our findings indicate that IV iron therapy resulted in a statistically significant greater median percentage increase in hemoglobin from POD3 to POD30, with a value of 5.93% (3.63–9.35%) in the IVI group compared to an increase in 3.58% (2.22–4.79%) in the control group. This finding is of significant importance, as it supports the use of IV iron postoperatively in anemic patients.

Furthermore, a subgroup analysis revealed that this effect was observed in the male patients (*p* = 0.004). In the female patients, although an increase was observed, it was not found to be statistically significant (*p* = 0.107). One potential explanation for this is that the study was underpowered to detect this effect. In addition, the IVI group exhibited a notable median percentage increase in hematocrit levels from POD3 to POD30, which further substantiates the efficacy of this intervention.

The findings of our study are in accordance with those of an RCT which showed that a single dose of IV ferric carboxymaltose was as effective in improving hemoglobin levels as multiple doses of iron sucrose IV [29]. Additionally, our results align with those of the retrospective study by the same research group in CRC patients [16]. This study also revealed a greater hemoglobin increment from POD1 to POD30 in postoperatively IV iron-treated patients compared to patients in the standard care group (oral or no iron therapy).

Moreover, the elevation in RBC count from POD3 to POD30 was not markedly superior in the IV iron group compared to the control group. This observation suggests that iron primarily stimulates hemoglobin production, while erythropoiesis typically requires a more extended timeframe. In both the PREVENTT trial and the RCT conducted by Khalafallah et al., the effects of IV iron (including increased hemoglobin, ferritin, and TSAT) were sustained at 8 weeks, 12 weeks, and even 6 months [15,17]. A limitation of our study is the relatively short follow-up period of 30 days.

A key aspect of our study was the stratification in patients with iron deficiency (either absolute or functional) and without iron deficiency on POD3 prior to IV iron administration. Iron deficiency was defined according to the criteria of ferritin concentration of less than 100 ng/mL and TSAT < 20%.

The percentage increase in hemoglobin was found to be statistically significantly greater in the IV iron group than in the control group among patients with iron deficiency. (*p* = 0.006), whereas in patients without iron deficiency, the increase was not statistically significant. A similar effect was observed in the HCT percentage change between the two groups. These findings are supported by the results of the PREVENT trial, which demonstrated that anemia was corrected at a higher rate in patients with iron deficiency compared to those without iron deficiency (risk ratio 2.06, 95% CI 1.27–3.35) [17]. This suggests that baseline iron status is a critical factor in determining the efficacy of IV iron therapy.

A closer examination of iron parameters revealed that the percentage change in ferritin and TSAT levels between POD3 and POD30 was significantly greater in the IVI group compared to the control group, irrespective of whether the patients were iron-deficient or non-iron-deficient. These results align with those of the aforementioned RCT for postoperative anemia, which demonstrated higher ferritin, iron, and TSAT levels in the ferric carboxymaltose group compared to the standard care group [15]. This was a significant finding not only at 4 weeks postintervention but also at 12 weeks. These findings indicate that IV iron therapy administered early postoperatively may be an effective intervention, even in the presence of adequate iron stores but suboptimal iron availability for erythropoiesis.

Regarding transfusions, only five (6.4%) patients received intraoperative RBC transfusions. There was no significant difference between the IVI group and the control group in the incidence of intraoperative transfusion. Nevertheless, transfusion in elective CRC surgery is relatively uncommon due to minimal intraoperative blood loss (6.4% blood loss >200 mL in our study) [6,12].

No serious adverse effects associated with the infusion of ferric carboxymaltose were recorded, which is being confirmed by the existing literature [12].

There were also some notable differences between the groups. The IV iron group had a significantly longer hospital stay of one additional day. This may be attributed to the closer monitoring and additional care required to manage IV iron administration and any associated complications. Secondly, with regard to postoperative complications, the IV iron group had more complications overall, with 38.9% of patients experiencing complications compared to 19% in the control group. However, this difference was predominantly attributable to the higher incidence of Clavien–Dindo grade I complications (27.8% vs. 7.1%). This higher rate of minor complications in the IV iron group could be due to more vigilant monitoring and reporting of side effects related to IV iron administration.

The decision to administer IV iron on POD3 was clinically and physiologically justified, as it addressed postoperative anemia in a timely manner. It is important to note that postoperative hemoglobin levels may vary due to various reasons such as fluid shifts, inflammation, and perioperative transfusions. It is known that within the initial 48 h following surgery, the body experiences a substantial neurohumoral stress response that includes renal vasoconstriction and the physiological conservation of salt and water [30,31]. We chose to administer IV iron on POD3 to allow the stress response to subside in a more stable physiological environment, thereby enabling us to address postoperative anemia at the earliest possible opportunity, in accordance with the guidelines [27].

The timing of IV iron therapy in CRC surgical patients remains a crucial aspect that requires further investigation. The present study concentrated on the efficacy and safety of postoperative administration. However, there is substantial evidence regarding the correction of anemia in the preoperative setting. A recent meta-analysis of five RCTs on preoperative administration of IV iron in CRC patients demonstrated that this approach resulted in elevated hemoglobin levels and a reduction in the necessity for allogeneic RBC transfusion without affecting the incidence of postoperative complications or the length of hospital stay [11]. Nevertheless, preoperative optimization is not always effectively organized, needing an additional hospital appointment, in health systems that are constrained by underfunding and understaffing [32]. In practice, patients are not optimized in the preoperative setting, resulting in a high prevalence of postoperative anemia in CRC patients (up to 87%), which in turn leads to worse outcomes [13]. Our findings suggest that administering IV iron on POD3 can effectively increase hemoglobin and tackle anemia, particularly in patients with documented iron deficiency. This suggests a potential benefit of early postoperative iron supplementation in anemic patients who have not been adequately treated preoperatively. The treating of anemia in the postoperative period offers additional benefits, including more efficient logistics, the management of patients with significant blood loss, and a more comprehensive assessment of the patient’s anemia by the surgeon [15].

The evidence currently available is insufficient to permit the establishment of a recommendation for the use of IV iron in the postoperative setting in CRC patients yet. Future studies should directly compare the efficacy of preoperative versus postoperative IV iron administration to further elucidate the optimal timing for intervention in CRC patients.

This study is subject to certain limitations. Firstly, the relatively small sample derived from a single center limits the power to detect differences and generalize the results. Although the two groups were matched on baseline characteristics, the non-randomized, non-blinded observational design may have induced bias in terms of considering potential confounders that may influence outcomes. These include surgeon preference in the allocation of patients to the treatment and control groups. Although we attempted to control for known confounding factors through statistical adjustments (subgroup analysis for male and female), the possibility of residual confounding factors cannot be eliminated and could be better addressed with a randomized controlled trial. Additionally, differences in postoperative care protocols (e.g., ERAS) and monitoring may influence the length of stay and complication reporting.

## 5. Conclusions

The findings of this study indicate that IV iron therapy administered at POD3 may be beneficial in enhancing hemoglobin levels by POD30 in anemic CRC patients, particularly in those with a documented iron deficiency. The considerable enhancements in ferritin and TSAT ratio provide further evidence of the efficacy of IV iron therapy. Consequently, patients with iron-deficiency anemia that was not corrected in the preoperative setting may benefit from an IV iron infusion in the early postoperative period, as this is an efficacious, safe, and expedient method of correcting postoperative anemia. Nevertheless, further large-scale, randomized controlled trials are required to validate these findings and to propose a treatment protocol for this group of patients.

## Figures and Tables

**Table 1:** Baseline characteristics, perioperative and postoperative data and outcomes.

	Total Number Ν = 78	Control Group Ν = 42	IV Iron Group Ν = 36	*p*-Value
Sex, Ν (%)				*p* = 0.249
Male	40 (51.3%)	19 (45.2%)	21 (58.3%)	
Female	38 (48.8%)	23 (54.8%)	15 (41.7%)	
Age (years), mean (±SD)	65.58 (±10.62)	67.50 (±9.61)	63.33 (±11.42)	*p* = 0.084
Body mass index (BMI) (kg/m^2^), mean (±SD)	27.46 (±5.05)	27.22 (±5.16)	27.74 (±4.98)	*p* = 0.652
ASA * physical status classification, Ν (%)				*p* = 0.716
I	32 (41.0%)	19 (45.2%)	13 (36.1%)	
II	36 (46.2%)	18 (42.9%)	18 (50%)	
III	10 (12.8%)	5 (11.9%)	5 (13.9%)	
Neoadjuvant therapy, Ν (%)				*p* = 0.363
No	64 (82.1%)	36 (85.7%)	28 (77.8%)	
Yes	14 (17.9%)	6 (14.3%)	8 (22.2%)	
Blood loss >200 mL, Ν (%)				*p* = 0.657
No	75 (93.6%)	40 (95.2%)	33 (91.7%)	
Yes	5 (6.4%)	2 (4.8%)	3 (8.3%)	
Intraoperative transfusion, Ν (%)				*p* = 0.657
No	73 (93.6%)	40 (95.2%)	33 (91.7%)	
Yes	5 (6.4%)	2 (4.8%)	3 (8.3%)	
Operative time (min), median (IQR)	190 (180.0–250.0)	180.0 (180.0–250.0)	205.0 (180.0–267.5)	*p* = 0.655
ICU admission, Ν (%)				*p* > 0.999
No	76 (97.4%)	41 (97.6%)	35 (97.2%)	
Yes	2 (2.6%)	1 (2.4%)	1 (2.8%)	
Clavien–Dindo, N (%)				***p* = 0.041**
No complications	56 (71.8%)	34 (81.0%)	22 (61.1%)	
1	13 (16.7%)	3 (7.1%)	10 (27.8%)	
2	8 (10.3%)	5 (11.9%)	3 (8.3%)	
3A	1 (1.3%)	0 (0.0%)	1 (2.8%)	
Length of stay, median (IQR)	6.0 (5.0–8.0)	6.0 (4.0–7.0)	7.0 (6.0–8.0)	***p* = 0.011**
Readmission, Ν (%)				*p* > 0.999
No	75 (96.2%)	40 (95.2%)	35 (97.2%)	
Yes	3 (3.8%)	2 (4.8%)	1 (2.8%)	

* ASA = American Society of Anesthesiology. The *p* values of the significant results are shown in bold.

**Table 2:** Male and female hemoglobin levels in male and female subjects in both groups, recorded at three time points: prior to surgery, at POD3, and at POD30.

Hemoglobin Level (g/dL)	Control Group		IVI Group		*p*-Value
	N	Mean (±SD) or Median (IQR)	N	Mean (±SD) or Median (IQR)	
Male					
Preoperative	19	13.11 (±1.74)	21	12.62 (±1.79)	*p* = 0.387
Postoperative 3rd day	19	11.25 (±1.50)	21	10.77 (±1.39)	*p* = 0.303
Postoperative 30th day	19	12.63 (±1.24)	21	13.04 (±1.36)	*p* = 0.309
Female					
Preoperative	23	12.55 (±1.92)	15	12.76 (±1.25)	*p* = 0.680
Postoperative 3rd day	23	10.68 (±1.71)	15	11.11 (±0.99)	*p* = 0.256
Postoperative 30th day	23	12.05 (±1.42)	15	12.96 (±1.07)	*p* = 0.042

**Table 3:** Changes in hematological and iron parameters according to iron deficiency status.

Hematologic Markers	Iron Deficiency	Control Group		IVI Group		*p*-Value
		N	Mean (±SD) or Median (IQR)	N	Mean (±SD) or Median (IQR)	
Percentage Hb change POD3 to POD30 (%)	Νο	8	4.19 (2.73, 9.11)	5	6.29 (3.79, 9.41)	*p* = 0.622
	Yes	24	3.36 (1.72, 4.51)	29	5.59 (3.67, 9.69)	*p* = 0.006
Percentage HCT change POD3 to POD30 (%)	Νο	8	17.16 (7.61, 25.78)	5	23.43 (4.41, 30.31)	*p* = 0.833
	Yes	24	10.75 (6.45, 18.41)	29	18.89 (9.57, 28.48)	*p* = 0.056
Percentage Ferritin change POD3 to POD30 (%)	Νο	8	−73.54 (−81.93, −56.66)	5	228.18 (106.68, 765.11)	*p* = 0.002
	Yes	23	−68.47 (−85.58, −42.03)	26	99.65 (17.94, 199.25)	*p* < 0.001
TSAT (%)						
POD3	Νο	8	21.44 (20.46, 22.31)	5	26.67 (21.67, 32.98)	*p* = 0.284
	Yes	24	12.01 (±4.11)	29	11.79 (±4.75)	*p* = 0.861
POD30	Νο	7	21.21 (12.24, 30.79)	5	41.95 (34.78, 50.90)	*p* = 0.030
	Yes	23	14.88 (11.00, 28.35)	26	32.64 (25.69, 38.33)	*p* < 0.001

## Data Availability

The original contributions presented in the study are included in the article, further inquiries can be directed to the corresponding author.

## Appendix A

**Table A1:** Analytic data presented for lab values observed at different time points.

Hematologic Markers	Control Group		IVI Group		*p*-Value
	N	Mean (±SD) or Median (IQR)	N	Mean (±SD) or Median (IQR)	
Hematocrit (%)—male					
Preoperative	19	40.63 (±4.71)	21	39.41 (±4.80)	*p* = 0.387
Postoperative 3rd day	19	34.72 (±4.22)	21	32.75 (±4.17)	*p* = 0.303
Postoperative 30th day	19	39.07 (±3.53)	21	39.95 (±3.90)	*p* = 0.308
Hematocrit (%)—female					
Preoperative	23	40.30 (35.00, 43.20)	15	41.00 (37.70, 41.70)	*p* = 0.658
Postoperative 3rd day	23	33.00 (29.00, 36.00)	15	35.00 (32.40, 36.00)	*p* = 0.497
Postoperative 30th day	23	38.20 (33.20, 40.60)	15	40.40 (36.60, 41.70)	*p* = 0.129
Ferritin—male (ng/mL)					
Preoperative	14	73.00 (34.25, 125.75)	15	32.00 (14.00, 70.00)	*p* = 0.588
Postoperative 3rd day	14	226.50 (174.00, 335.75)	15	116.00 (79.00, 179.00)	*p* = 0.005
Postoperative 30th day	14	62.65 (37.75, 104.33)	15	212.80 (124.00, 320.00)	*p* < 0.001
Ferritin—female (ng/mL)					
Preoperative	17	44.00 (22.00, 102.00)	14	75.50 (16.75, 228.50)	*p* = 0.610
Postoperative 3rd day	17	161.00 (107.00, 194.00)	14	150.50 (100.50, 247.75)	*p* = 0.682
Postoperative 30th day	17	58.00 (18.00, 111.50)	14	366.70 (254.00, 526.00)	*p* < 0.001
Folic acid (ng/mL)					
Preoperative	28	6.65 (4.48, 7.98)	27	6.00 (3.40, 8.70)	*p* = 0.569
Postoperative 3rd day	28	5.75 (4.05, 8.48)	27	5.00 (3.40, 7.50)	*p* = 0.214
Postoperative 30th day	28	5.45 (3.70, 8.10)	27	4.80 (3.80, 7.70)	*p* = 0.441
Β12 (pg/mL)					
Preoperative	28	331.50 (275.00, 449.25)	28	374.50 (255.50, 445.00)	*p* = 0.516
Postoperative 3rd day	28	453.00 (247.25, 553.75)	28	359.50 (275.00, 506.25)	*p* = 0.470
Postoperative 30th day	28	352.00 (283.63, 428.00)	28	361.00 (254.50, 489.25)	*p* = 0.284
INR					
Preoperative	25	1.03 (±0.11)	30	1.01 (±0.08)	*p* = 0.332
Postoperative 3rd day	25	1.05 (±0.08)	30	1.06 (±0.10)	*p* = 0.746
Postoperative 30th day	25	1.09 (±0.26)	30	1.02 (±0.11)	*p* = 0.171
Fibrinogen (mg/dL)					
Preoperative	26	436.59 (±107.52)	29	402.36 (±126.71)	*p* = 0.288
Postoperative 3rd day	26	538.05 (478.35, 621.23)	29	539.00 (503.40, 561.10)	*p* = 0.474
Postoperative 30th day	26	383.75 (354.53, 427.60)	29	395.60 (327.00, 440.45)	*p* = 0.174
APTT (sec)					
Preoperative	26	28.56 (±4.25)	30	29.34 (±2.88)	*p* = 0.569
Postoperative 3rd day	26	30.22 (±4.45)	30	31.41 (±3.95)	*p* = 0.562
Postoperative 30th day	26	29.85 (±3.35)	30	30.19 (±3.69)	*p* = 0.770
PLT (1000 cells/μL)					
Preoperative	42	240.16 (±76.76)	36	222.11 (±76.74)	*p* = 0.304
Postoperative 3rd day	42	208.99 (±77.22)	36	193.94 (±68.22)	*p* = 0.368
Postoperative 30th day	42	251.66 (±77.81)	36	227.63 (±71.33)	*p* = 0.162
CRP (mg/dL)					
Preoperative	40	0.20 (0.10, 0.50)	34	0.20 (0.10, 0.55)	*p* = 0.771
Postoperative 3rd day	40	8.65 (4.10, 12.80)	34	8.45 (4.05, 12.23)	*p* = 0.988
Postoperative 30th day	40	0.20 (0.10, 0.78)	34	0.16 (0.10, 0.66)	*p* = 0.667
